# Assessing Microcirculation in Heart Failure: No Sweet Without Sweat

**DOI:** 10.1016/j.jacadv.2026.102751

**Published:** 2026-04-22

**Authors:** Christian Jung, Sabri Soussi, Maryna Masyuk

**Affiliations:** a Department of Cardiology, Pulmonology and Vascular Medicine, University Hospital and Medical Faculty, Heinrich-Heine University, Duesseldorf, Germany; b Cardiovascular Research Institute Düsseldorf (CARID), Medical Faculty, Heinrich-Heine University, Duesseldorf, Germany; c Department of Anesthesiology and Pain Medicine, University of Toronto, Toronto, Ontario, Canada; d Inserm UMR-S 942, Cardiovascular Markers in Stress Conditions (MASCOT), University of Paris, Paris, France

**Keywords:** heart failure, microcirculation, sublingual microscopy

**Figure:**
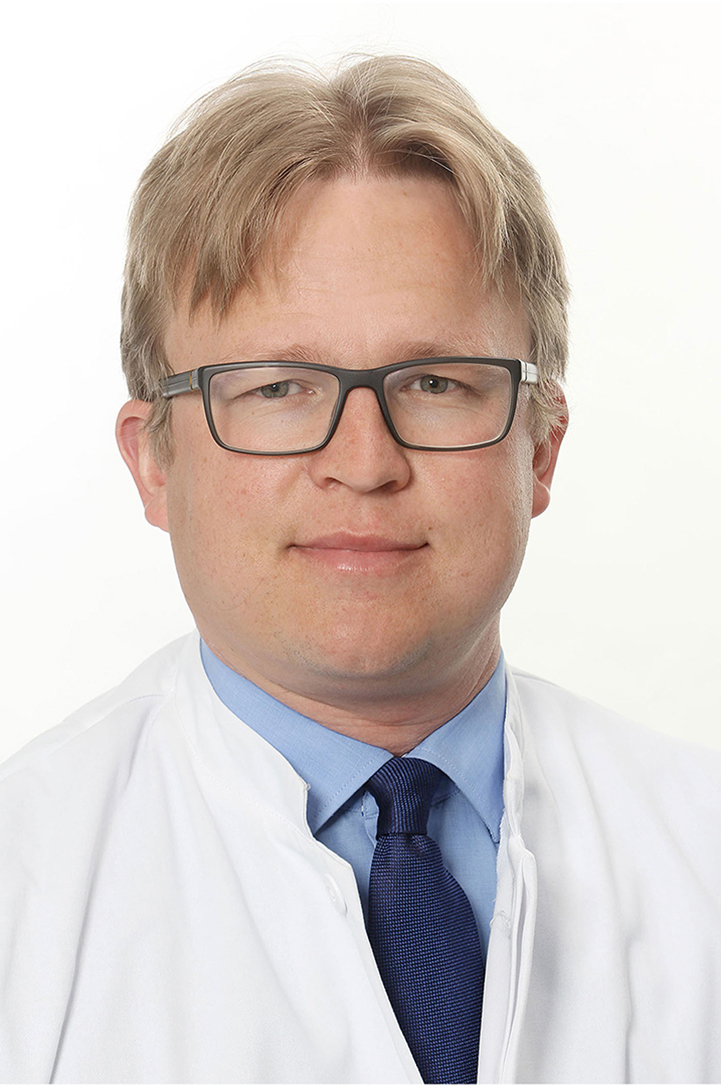

In the last decades, we have learned that restoration of systemic hemodynamic parameters in critically ill patients does not always lead to normalization of microcirculation, which itself is crucial for tissue oxygen supply and homeostasis.^1^ An incoherence between macrocirculatory and microcirculatory state of the patients is associated with adverse outcome.^2^ Therefore, assessment of microcirculatory function in different cohorts of shock and heart failure has shifted into focus of research and clinicians. In this issue of *JACC: Advances*, Baranowska et al.^3^ present a study comparing microcirculatory parameters derived from sublingual microscopy between different groups of heart failure patients, ranging from stable chronic heart failure patients to patients in acute cardiogenic shock, recipients of left ventricle assist device (LVAD) support or patients after heart transplantation. The authors describe considerable differences between the groups and report a particularly impaired microcirculation in patients in cardiogenic shock and after heart transplantation whereas patients with chronic heart failure and LVAD-support displayed better microcirculatory parameters. It is important to congratulate the authors for their efforts to create a comprehensive comparison across disease severity states in heart failure by prospectively recruiting a large number of patients with a homogenous recruitment strategy. Based on these tremendous efforts we would like to contextualize the findings.

## Microcirculatory assessment by sublingual microscopy: methods and evidence

When interpreting the results of the present study, we first have to consider the methodology used to quantify “microcirculation”. Based on the intensive work conducted by several working groups in the field of sublingual microcirculation, the European Society of Intensive Care Medicine has released a consensus statement to provide a guideline for clinicians and scientists using hand-held vital microscopes.^4^ To analyze sublingual microcirculation, different approaches are recommended. Ideally, a detailed manual assessment of functional parameters, such as De Backer Score, based on calculating vessel density by vessel crossings through a predefined grid on the screen, with consequent calculation of proportion of perfused vessels (and perfused vessel density, or microvascular flow index should be performed.^5^ This can be complemented by software-based analyses, such as automated vessel analysis, although limitations exist. In the present study, the authors have used the GlycoCheck software, a software with parameters claiming to describe microcirculatory status of the patients by estimating the glycocalyx’ thickness in sublingual vessels by using perfused boundary region.^6^ The authors provide several parameters, such as microvascular health score or capillary blood volume calculated by the software. When looking into the literature, there are studies using parameters derived from GlycoCheck software in different clinical settings; however, they have been poorly validated against established sublingual microcirculatory parameters: for instance, a study involving 30 patients with sepsis and 10 healthy volunteers has found no correlation between perfused boundary region and established sublingual microcirculatory parameters.^7^ Moreover, the reported measurement durations with the applied technology ranging from 10 to 90 minutes, seem to be impracticable for clinical use and may affect the image quality. Especially pressure artefacts are probably inevitable in such long recording times and should be systematically addressed using the recommended microcirculation-imaging-quality-score).^8^ These methodological approach makes it difficult to directly compare and to interpret.

## Sublingual microcirculation in heart failure

Baranowska et al have undertaken an enormous effort to recruit a very large cohort of patients, with 376 heart failure patients and 44 controls. In the past, several studies have investigated sublingual microcirculation in stable heart failure, cardiogenic shock, or ventricular assist devices,^9^, ^10^, ^11^ including a randomized controlled multicenter trial using sublingual microcirculatory parameters to guide therapy management in shock patients.^12^ Impaired microcirculation in shock seems to be a hallmark phenomenon. In the present work, the authors present a comparison of different states of heart failure. They show, that indeed stable heart failure patients and patient on LVAD support show impaired microcirculatory parameters as compared to controls, which is, however, considerably better than in cardiogenic shock patients. This is a definite forte of this work, which directly allows us to observe a gradual decline of microcirculatory function along the different entities of heart failure. However, a matched control cohort regarding age and noncardiac comorbidities would help to eliminate other confounders than heart failure contributing to impaired sublingual microcirculation. Moreover, information regarding cardiogenic shock patients in terms of vasopressor use and parameters reflecting the severity of hypoperfusion such as lactate levels and lactate kinetics would allow to set the results in a proper context. Furthermore, the present study is the first systematic study analyzing microcirculation in heart transplantation recipients. The results are very interesting and surprising as they show an altered microcirculatory status in these patients despite normalized cardiac output. At this stage, this should be interpreted as hypothesis-generating pending confirmation by other hardware solutions or strategies to investigate the microcirculation.

## Future perspectives

One of the main obstacles to implement sublingual microcirculation assessment into clinical everyday practice is the time-consuming analysis, even by the use of an automated software. Therefore, the European Society of Intensive Care Medicine experts have already stated the need of new automated and validated software in their second consensus statement as prerequisite for the improvement of the hand-held vital microscopes. In the present work, Baranowska et al. use a novel software in a very large and prospectively enrolled patient cohort, providing very interesting results. However, the software parameters are not adequately validated to surely reflect microcirculatory dysfunction. To understand whether this software might represent a progress in sublingual microcirculatory assessment, validation studies with systematic comparisons with internationally recognized and established sublingual microcirculation parameters are warranted. Such systematic validation of the software and also a systematic analysis of different heart failure entities using established and novel methods of sublingual microcirculatory analysis in combination with other microcirculatory parameters, such as lactate levels, lactate kinetics, capillary refill time, as well as other methods assessing peripheral tissue perfusion, for example peripheral near-infrared spectroscopy, and endothelial injury biomarkers, would be a major effort, but would certainly substantially contribute to our understanding of microcirculation in heart failure. It would moreover allow us to identify and validate novel techniques for sublingual microcirculatory assessment and possibly implement it in the clinical daily practice. Besides that, the sustained microcirculatory impairment in patients after heart transplantation despite normalized microcirculatory parameters is a very interesting finding, which needs confirmation with established methods and, if reproducible, is of great interest for future mechanistic studies. Microcirculation assessment has the potential to improve and guide therapy for individualized care. Before this can be achieved, we need to shape the understanding of the microcirculation and harmonize how we determine it.

## Funding support and author disclosures

The authors have reported that they have no relationships relevant to the contents of this paper to disclose.
